# A New Ratiometric Fluorescent Probe Based on BODIPY for Highly Selective Detection of Hydrogen Sulfide

**DOI:** 10.3390/molecules27217499

**Published:** 2022-11-03

**Authors:** Huan Xiang, Tianqing Ye, Yanbo Li, Yanfei Lin, Dan Liu, Hongwei Zhou, Jianbo Wang, Lei Li

**Affiliations:** Jiaxing Key Laboratory of Molecular Recognition and Sensing, College of Biological, Chemical Sciences and Engineering, Jiaxing University, Jiaxing 314001, China

**Keywords:** hydrogen sulfide, BODIPY, ratiometric fluorescent probe, nucleophilic reaction, colorimetric

## Abstract

Hydrogen sulfide (H_2_S) as small molecular signal messenger plays key functions in numerous biological processes. The imaging detection of intracellular hydrogen sulfide is of great significance. In this work, a ratiometric fluorescent probe **BH** based on an asymmetric BODIPY dye for detection of H_2_S was designed and synthesized. After the interaction with hydrogen sulfide, probe display colorimetric and ratiometric fluorescence response, with its maximum emission fluorescence wavelength red-shifted from 542 nm to 594 nm, which is attributed to the sequential nucleophilic reaction of H_2_S leading to enhanced molecular conjugation after ring formation of the BODIPY skeleton. A special response mechanism has been fully investigated by NMR titration and MS, so that the probe has excellent detection selectivity. Furthermore, probe **BH** has low cytotoxicity and fluorescence imaging experiments indicate that it can be used to monitor hydrogen sulfide in living cells.

## 1. Introduction

As the third gaseous transmitter after nitric oxide (NO) and carbon monoxide (CO), hydrogen sulfide (H_2_S) has gained more attention due to its multiple features in biological progresses [1,2,3]. Intracellular H_2_S is generated by enzymes such as cysteine gamma-lyase (CSE) and cysteine beta synthase (CBS) catalyzed by sulfur-containing amino acids, and further plays important roles in regulation of several physiological activities, such as vascular tone, neuromodulation, inflammation and apoptosis [4,5,6]. Abnormal concentrations of H_2_S are often correlated with several diseases including diabetes, Alzheimer’s and liver cirrhosis [7,8,9]. Hence, it is of significance to monitor the intracellular H_2_S levels for thorough understanding of the H_2_S functional roles in living cells.

Conventional tools including colorimetric method, chromatography and electrochemical methods are difficult to monitor the intracellular H_2_S because of their shortcomings, such as low sensitivity and complicated operation [10,11,12]. Fluorescence probe is regarded as one of the most powerful means for detecting analysis and visualizing imaging of biologically relevant species due to the high sensitivity, spatial–temporal resolution and real-time imaging [13,14]. Among numerous fluorophores, Boron dipyrromethenes (BODIPY) dyes have been widely applied in fluorescence sensing and imaging because of their high absorption coefficient, excellent fluorescent quantum yield and high photo-stability [15,16]. So far, tremendous efforts have been devoted to the exploitation of BODIPY-based fluorescent probes for monitoring and imaging of endogenous hydrogen sulfide in living cells with high sensitivity and low detection limits [17,18,19,20,21,22,23,24,25,26,27,28]. However, most of the reported H_2_S fluorescent probes based on BODIPY are focused on the “turn-on” or “turn-off” detection model in which the fluorescence intensity of probes increases with the concentration of hydrogen sulfide. However, the single fluorescent intensity change is also easily limited to the interference of the environment factors, excitation light source and probe concentration. The ratiometric fluorescent probe exhibits good detection accuracy through the ratio of fluorescent intensity at two channel wavelengths to avoid the systemic error in biological imaging. In addition, the key to the design strategy of the H_2_S fluorescence probe relies on the reaction of the probe with hydrogen sulfide including the nucleophilic reaction, Michael addition reaction, reduction of nitro or azide and metal ion complex type [29,30,31,32,33,34,35]. The nucleophilic addition reaction of hydrogen sulfide with a probe triggers the fluorescence recovery through changing the intramolecular charge transfer or blocking the photoinduced electron transfer process. However, this single-nucleophilic reaction is easily interfered by cellular active sulfur sources (cysteine and glutathione), resulting in poor sensing selectivity. Hence, the development of a fluorescent probe based on a BODIPY scaffold that exhibits ratiometric fluorescent intensity change toward H_2_S with excellent detection selectivity remains a challenge.

**Scheme 1:**
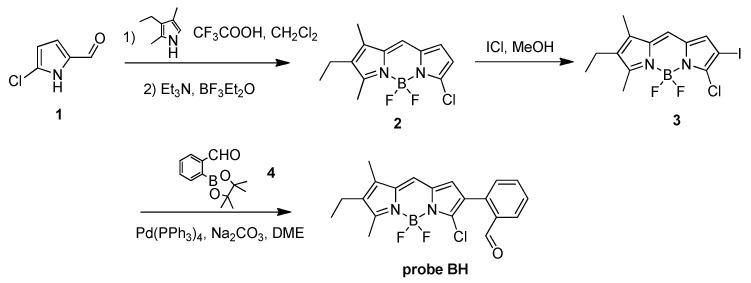
Synthetic routes of fluorescent probe **BH**.

In this work, we developed a new ratiometric fluorescence sensor for excellent detection of hydrogen sulfide via the sequential-nucleophilic addition reaction. The BODIPY fluorophore was chosen to implement this strategy due to its good photo-properties in fluorescence sensing and imaging. Importantly, the chlorine substituted in 3-position of BODIPY has been designed to turn-on the fluorescent probe for the monitoring of H_2_S or bio-thiol based on the nucleophilic reaction. Another critical group which is easily attacked by nucleophilic agent is the aldehyde group. In view of these considerations, we introduced 2-formaldehyde phenyl and chlorine as two reaction sites in 2,3-position of BODIPY dyes to construct the probe BH. As Scheme 1 show, two reaction sites of the probe were continuously attacked by H_2_S, giving 2,3-position closing-ring products with the molecular π-conjugation expansion, and resulting in the fluorescence emission red-shift from 542 nm to 594 nm. This double nucleophilic reaction of response feature makes the probe display ratiometric fluorescence sensing and high selectivity toward H_2_S. Finally, the probe is capable of ratiometrically monitoring H_2_S changes in living cells.

## 2. Results and Discussion

### 2.1. Design and Synthesis of Probe **BH**

We reported on a ratiometric fluorescent probe based on the BODIPY dye for the monitoring of Zn^2+^ through regulating the intramolecular charge transfer [36]. Considering the double nucleophilic feature of hydrogen sulfide, two response sites (2-formaldehyde phenyl and chlorine) were introduced into the 2,3-positions of BODIPY fluorophore to design the ratiometric fluorescent probe **BH**, which achieves a spectral red-shift by expanding the molecular conjugation system after reacting with hydrogen sulfide to form a ring. As shown in Scheme 1, the important compound 3-chloro substituent BOIDPY was easily obtained by the asymmetry BODIPY methods. Iodination reaction with ICI and Suzuki reaction with 2-formaldehyde phenylboronic was subsequently carried out to successfully obtain probe **BH** in an acceptable yield. The intermediates and probe were fully characterized by NMR and mass spectrometry.

### 2.2. Spectral Response of Probe **BH** toward H_2_S

**Figure 1:**
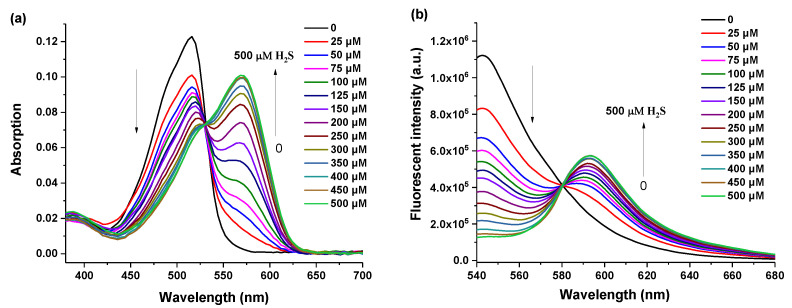
The absorption (**a**) and fluorescence (**b**) spectrum changes of probe **BH** (5 μM) toward different concentration of H_2_S (0–500 μM) in aqueous solution of DMSO/PBS (*v*/*v* 1/1, pH 7.4), recorded after 60 min.

**Figure 2:**
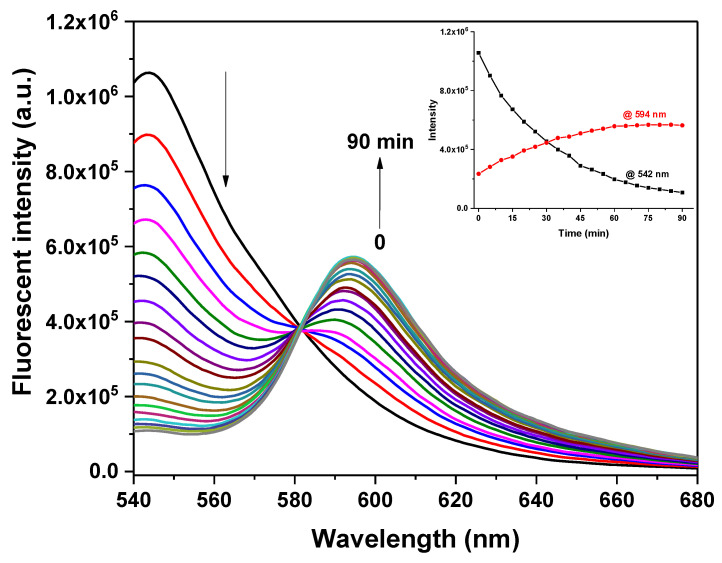
Time-dependent fluorescence specturm changes of probe **BH** (5 μM) toward H_2_S (500 μM) in aqueous solution of DMSO/PBS (*v*/*v* 1/1, pH 7.4); inset: fluorescence intensity at 594 nm and 542 nm as a function of detection time.

In testing the solution for DMSO/PBS (*v*/*v*, 1:1, pH 7.4), probe BH displays a strong and narrow absorption in the visible region with a peak at 516 nm, which is characterized by BODIPY dyes. After the addition of different concentrations of H_2_S, the absorption spectrum is obviously red-shifted and accompanied by the maximum absorption peak shifted to 568 nm with the iso-absorption points as 530 nm, reaching saturation at 100 eq. H_2_S (Figure 1a and Figure S1). This change is accompanied by the color change of the solution from orange to purple. At the same time, the fluorescence spectrum shows the corresponding change. As shown in Figure 1b, probe BH exhibits the strong fluorescence emission with peak at 542 nm. With the gradual addition of H_2_S, the fluorescence emission band of solution obviously shifts and a new peak at 594 nm was formed. The drastic fluorescence changes in two wavelengths (594 nm and 542 nm) brings about a 18-fold enhancement of intensity ratio from 0.217 to 4.107 in the presence of 500 μM H_2_S (Figure S2). The fluorescence ratio of a time-dependent probe BH was also studied in the presence of 500 μM H_2_S and the results are shown in Figure 2. The emission intensity of the probe at both 594 nm and 542 nm reaches a relatively saturated stage within 60 min. In addition, the fluorescence intensity ratio (I_594nm_/I_542nm_) of the probe displayed a linear relationship variation in a concentration range of H_2_S (0–75 µM) (Figure S3). So, the detection limit of the probe toward H_2_S was calculated as 3.5 µM based on the conventional 3 σ/k method, indicating that BH is sensitive to H_2_S.

### 2.3. Response Mechanism Studies

**Figure 3:**
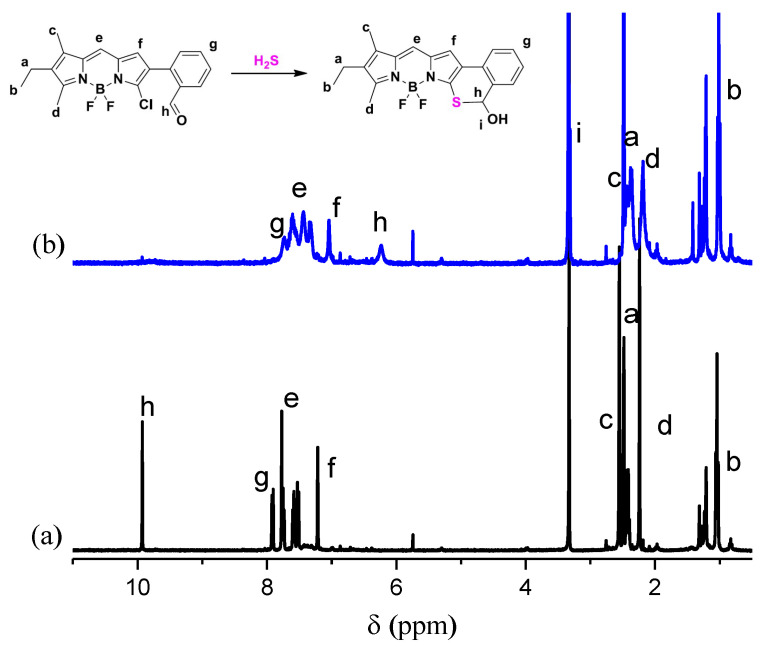
The ^1^H-NMR titration of probe **BH** toward H_2_S in DMSO-d6 (400 MHz), (**a**) **BH**; (**b**) **BH** + H_2_S.

**Scheme 2:**
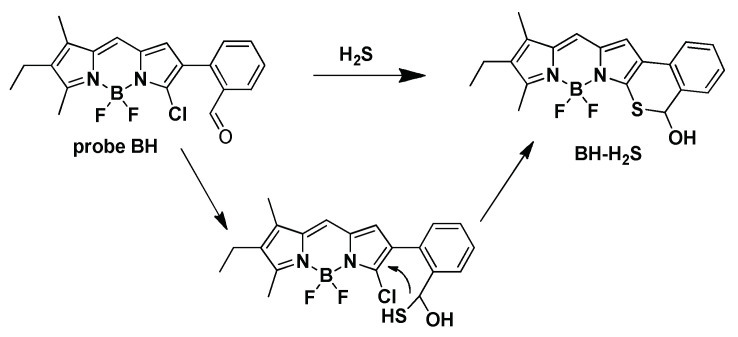
The proposed sensing mechanism of probe **BH** responses toward H_2_S.

The intensive colorimetric and dual-channel fluorescence with a red-shift change of probe toward hydrogen sulfide led us to explore the response mechanism. ^1^H-NMR titration and MS spectroscopy were used to investigate the response products. As shown in Figure 3, with the addition of H_2_S, the disappearance of signal proton of aldehyde hydrogen and the appearance of two new sets of signals at 5.0 and 3.3 ppm indicate that the aldehyde group of the probe undergoes a nucleophilic reaction with H_2_S. The protons of BODIPY skeleton and 2-phenyl moiety also display high-field shifts, suggesting the binding of S atom to the 3-position of BODIPY. These results are also confirmed by the mass spectrometry of probe **BH** after the addition of H_2_S. A mass peak at *m*/*z* = 383.1180 in Figure S4 is the mass spectral signal of the response product **BH**-H_2_S ([M-H]^−^, calculated at 383.1199). Hence, the fluorescence ratiometric response of the probe toward H_2_S is attributed to the formation of ring structure in the BODIPY skeleton derived from the sequential nucleophilic reaction of hydrogen sulfide with the chlorine and benzaldehyde groups of the probe, resulting in enhanced molecular conjugation and rigidity (Scheme 2). Most of reported BODIPY-based H_2_S probes are single-channel fluorescence Turn-On or OFF mode (Table S1), Our work provides the more reliable two-channel fluorescence change detection of hydrogen sulfide on BODIPY dyes.

### 2.4. Selectivity and pH Effect

**Figure 4:**
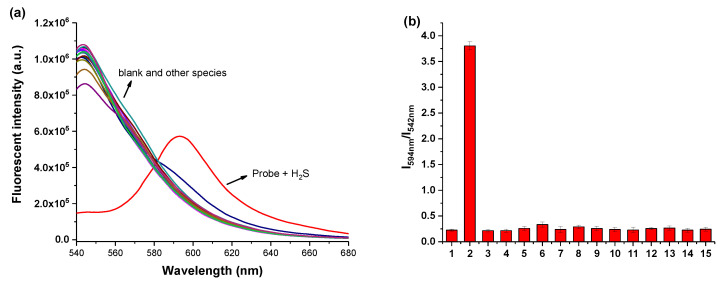
The fluorescence spectrum change (**a**) and intensity ratios (**b**) of probe **BH** (5 μM) in the presence of various species (100 eq.) in DMSO/PBS (*v*/*v* 1/1, pH 7.4), include 1. blank, 2. H_2_S, 3. Ala, 4. Glu, 5. GSH, 6. Cys, 7. Hcy, 8. H_2_O_2_, 9. S_2_O_3_^2−^, 10. Br^−^, 11. NO_2_^−^, 12. SO_4_^2−^, 13. ClO^−^, 14. Mg^2+^ and 15. K^+^, recorded after 60 min.

To evaluate the detection selectivity of the probe, various interference species including Ala, Glu, GSH, Cys, Hcy, H_2_O_2_, S_2_O_3_^2−^, Cl^−^, Br^−^, NO_2_^−^, SO_4_^2−^, ClO^−^, Mg^2+^ and K^+^ were treated with the probe in DMSO/PBS (*v*/*v*, 1:1, pH 7.4) and results are shown in Figure 4. These species, especially biothiols (Cys/Hcy/GSH), did not cause any significant change in the fluorescence intensity ratio (I_594nm_/I_542nm_). On the contrary, only hydrogen sulfide caused significant changes, indicating that the probe displays high selectivity towards hydrogen sulfide over other species. The detection of pH effect on the ratiometric fluorescent response of the probe toward hydrogen sulfide was also investigated. As shown in Figure S5, the probe showed a similar lower fluorescence ratio (I_594nm_/I_542nm_) in the absence of H_2_S, indicating the probe has good spectral stability in a pH range of 4–9. After treatment of H_2_S, a significant fluorescence ratio change was observed within a pH range of 6.4 to 9. Hence, the probe is suitable for monitoring of hydrogen sulfide in physiological conditions.

### 2.5. Probe Application in Cellular Imaging

**Figure 5:**
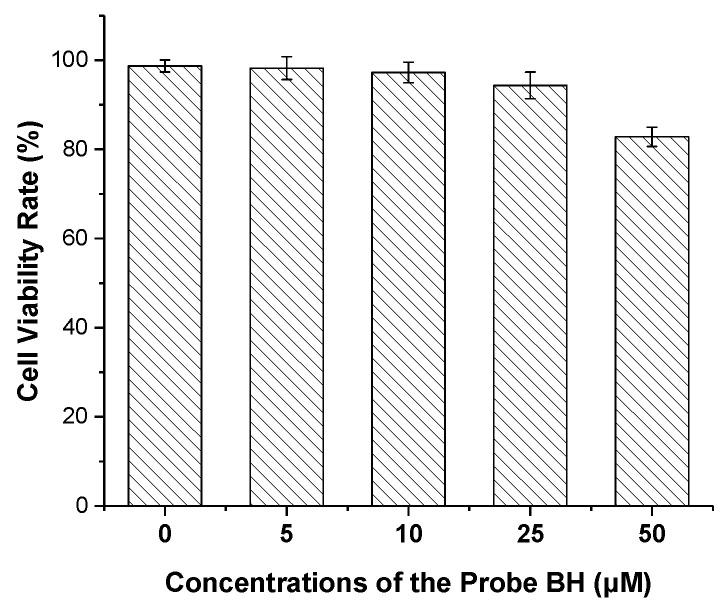
Cell viability values (%) estimated by an MTT assay versus incubation concentrations of CT26 cells were cultured in the presence of probe (0–50 μM) at 37 °C for 48 h.

**Figure 6:**
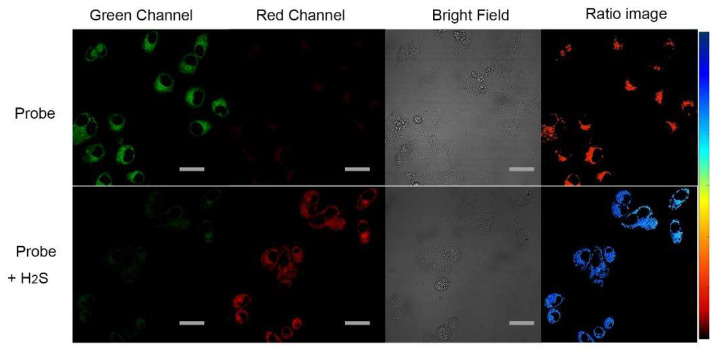
Confocal fluorescence images of Hela cells incubated with probe **BH** (5 μM) in the absence and presence of H_2_S (300 μM). The ratio image is based on the red channel divided by the green channel. Scale bar: 20 μm.

To explore the potential application of the probe in living cells, confocal fluorescence imaging experiments were performed. We first investigated the cytotoxicity of the probe using standard MTT assays. More than 90% of the cells were viable when incubated with a 25 µM probe for 24 h (Figure 5), indicating the very low cytotoxicity of probe. The cellular imaging of the probe was carried out for the monitoring of H_2_S in living cells. After the probe (5 µM) was incubated in Hela cells for 30 min, there was a strong fluorescent signal in the green channel (510–560 nm), while there was almost no signal in the red channel (570–630 nm). Once the cells were further incubated with H_2_S (300 µM) in culture medium for 60 min, the fluorescent signal in green channel was weakened while the emission signal in the red channel was significantly enhanced (Figure 6), indicating that the probe is able to monitor intracellular H_2_S in living cells.

## 3. Materials and Methods

### 3.1. Materials and Instrumentation

Unless otherwise stated, all chemicals were purchased from commercial sources and used as received without further purification. Solvents of technical quality were distilled prior to use. Double distilled water was used throughout the experiments. ^1^H and ^13^C NMR spectra were acquired over a Bruker AV spectrometer operating at 400 MHz and 100 MHz, respectively. Mass spectra were performed with a Micromass GCF TOF spectrometer. UV-Vis absorption and fluorescence emission spectra were obtained at room temperature with Agilent Cary 5000 UV–vis spectrometer and HORIBA Fluorolog-3 spectrophotometer, respectively.

### 3.2. Preparation of Sample Solutions

A stock solution of probe **BH** (1.25 mM) was prepared in DMSO. The sample probe (5 μM) solutions was obtained by adding 10 μL of probe stock solution using micro syringes into the 2.5 mL of DMSO/Phosphate buffer saline (PBS, pH 7.4, 20 mM, *v*/*v* 1:1) solution in quartz cuvette (1 cm × 1 cm). H_2_S solution was prepared by dissolution of NaHS in deionized water. After a certain amount of H_2_S solution (2.5 mM) was added and after waiting for 60 min, the absorption of the solution was recorded, and the fluorescence spectra was collected under the excitation of 530 nm. All samples and references were freshly prepared under similar conditions.

### 3.3. Synthesis

Synthesis of compound **2**. Three drops of TFA was added to the solution of 5-chloro-pyrrole-2-carbaldehyde (0.39 g, 3 mmol) and 2,4-dimethyl-3-ethylpyrrole (0.36 g, 3 mmol) in dry CH_2_Cl_2_ (80 mL) under N_2_. The reaction was stirred overnight at room temperature. Et_3_N (3 mL) and BF_3_ Et_2_O (3 mL) were successively added and the reaction was continually stirred for 2 h. Then, the mixture was washed with brine and an organic layer was dried over sodium sulfate, filtered and concentrated under vacuum. The residue was purified by flash column chromatography to give compound 2 (0.36 g, 43%). ^1^H NMR (400 MHz, CDCl_3_) δ: 6.98 (s, 1H), 6.79 (d, J = 3.6 Hz, 1H), 7.25 (d, J = 3.2 Hz, 1H), 2.58 (s, 3H), 2.43–2.37 (m, 2H), 2.17 (s, 3H), 1.08 (t, J = 3.6 Hz, 3H); ^13^C NMR (100 MHz, CDCl_3_) δ: 163.9, 140.9, 136.9, 136.0, 135.3, 131.6, 125.4, 122.1, 115.1, 17.2, 14.2, 13.3, 9.4.

Synthesis of compound **3** Compound 2 (0.28 g, 1 mmol) was dissolved in a mixture solution of N, N-dimethylformamide and methanol (5 mL/10 mL). Iodine monochloride (0.19 g, 1.2 mmol) was slowly added dropwise at room temperature and the reaction was stirred for 1 h. Sodium thiosulfate solution (20 mL) was used to quench the reaction and further extracted with dichloromethane (40 mL*2). The organic layer was dried with sodium sulfate, filtered, concentrated and further purified by silica gel column chromatography to obtain a red solid compound 3 (0.3 g, 74%). ^1^H NMR (400 MHz, CDCl_3_) δ: 6.92 (s, 1H), 6.88 (s, 1H), 2.58 (s, 3H), 2.40 (q, J = 7.6 Hz, 2H), 2.18 (s, 3H), 1.08 (t, J = 7.6 Hz, 3H); ^13^C NMR (100 MHz, CDCl_3_) δ: 166.1, 142.0, 138.9, 136.3, 132.5, 130.3, 120.9, 77.0, 17.3, 14.0, 13.3, 9.4.

Synthesis of probe **BH**. A solution of compound 3 (50 mg, 0.12 mmol) and compound 4 (32 mg, 0.13 mmol) in THF (10 mL) was bubbled with argon for 10 min, then, Pd(PPh_3_)_4_ (7 mg, 6 μmol) and K_2_CO_3_ (2 M, 0.2 mL) were added. The mixture was heated at 80 °C for 8 h. After cold to r.t, ethyl acetate (20 mL) and water (10 mL) was poured into the mixture. The organic layer was collected, dried with sodium sulfate, filtered and concentrated. The residue was purified by silica gel column chromatography to obtain probe BH as an orange solid (32 mg, 68%). ^1^H NMR (400 MHz, CDCl_3_) δ: 10.06 (s, 1H), 8.01 (d, J = 8.0 Hz, 1H), 7.63–7.65 (m, 1H), 7.47–7.52 (m, 2H), 7.06 (s, 1H), 6.85 (s, 1H), 2.63 (s, 3H), 2.42–2.47 (m, 2H), 2.22 (s, 3H), 1.12 (t, J = 8Hz, 3H); ^13^C NMR (100 MHz, CDCl_3_) δ: 192.0, 166.2, 141.7, 136.3, 134.0, 133.7, 131.4, 130.7, 128.2, 127.6, 125.1, 121.8, 17.3, 14.1, 13.6, 9.5; MS (ESI): calculated for C_20_H_18_BClF_2_NaO [M+Na]^+^ 409.1065, found 409.1060.

### 3.4. Probe Application in Cellular Imaging

The Hela cells were cultured in Modified Eagle medium containing 10% fetal bovine serum (FBS) under an atmosphere of 5% CO_2_ at 37 °C. The cell culture medium was replaced with a freshly FBS-free medium including probe BH, and then incubated for 30 min. For the imaging of H_2_S, the cells were further incubated with H_2_S (300 µM) for 60 min. Cells were washed three times with PBS buffers prior to imaging. A confocal fluorescence microscope (Olympus FV 1000) was used to collect cellular fluorescence images. The green channel and red channel emissions of the probe were collected in the emission range of 510–560 nm and 570–630 nm with excitation at 488 nm and 543 nm, respectively.

## 4. Conclusions

In summary, a new BODIPY-based probe with ratiometric fluorescent change for the monitoring of hydrogen sulfide was designed and synthesized. Due to the sequential nucleophilic substitution reactions with hydrogen sulfide at the 2,3-positions of the BODIPY fluorophore, the probe displayed fluorescence ratiometric detection with a red-shift in both the absorption and fluorescence emission. This specific response mechanism was fully characterized by ^1^H NMR and mass spectrometry and allowed the detection to exhibit excellent selectivity and sensitivity. In addition, the probe exhibited low cytotoxicity and was used to visualize intracellular H_2_S in living cells. This design strategy based on the enhanced molecular conjugation of the probe through the sequential nucleophilic reaction offers new opportunities for the construction of the ratiometric fluorescent probe toward hydrogen sulfide.

## Data Availability

The data that support the findings of this study are available from the corresponding author upon reasonable request.

## Supplementary Materials

The following supporting information can be downloaded at: https://www.mdpi.com/article/10.3390/molecules27217499/s1, Table S1: The BODIPY-based fluorescent probes for detection of H_2_S; Figure S1: The absorption ratio at 568 nm and 516 nm of probe BH (5 μM) toward different concentration of H_2_S in aqueous solution of DMSO/PBS (*v*/*v* 1/1, pH 7.4); Figure S2: The fluorescence intensity at 568 nm and 516 nm of probe BH (5 µM) toward increasing H_2_S concentration (0−500 μM) in aqueous solution of DMSO/PBS (*v*/*v* 1/1, pH 7.4); Figure S3: Linear relationship of the fluorescence intensity ratio (I_594nm_/I_542nm_) of probe BH toward H_2_S concentration (0−75 μM); Figure S4: ESI-MS spectrum of reaction product of probe BH with H_2_S; Figure S5: The fluorescence intensity ratios (I_594nm_/I_542nm_) of probe BH (5 μM) in the absence (●) or presence (■) of H_2_S (500 μM) at various pH values [18,19,20,22,23,24,25,26,27,28,37,38,39,40].

## Sample Availability

Samples of the compounds are available from the authors.

## References

[1] Culotta E., Koshland D.E. (1992). No news is good news: A startlingly simple molecule unites neuroscience, physiology, and immunology and revises. Science.

[2] Han Y., Qin J., Chang X., Yang Z., Du J. (2006). Hydrogen sulfide and carbon monoxide are in synergy with each other in the pathogenesis of recurrent febrile seizures. Cell. Mol. Neurobiol..

[3] Park C.S., Ha T.H., Choi S.-A., Nguyen D.N., Noh S., Kwon O.S., Lee C.-S., Yoon H. (2017). A near-infrared “turn-on” fluorescent probe with a self-immolative linker for the in vivo quantitative detection and imaging of hydrogen sulfide. Biosens. Bioelectron..

[4] Xuan W., Sheng C., Cao Y., He W., Wang W. (2012). Fluorescent probes for the detection of hydrogen sulfide in biological systems. Angew. Chem. Int. Ed..

[5] Wang R. (2012). Physiological implications of hydrogen sulfide: A whiff exploration that blossomed. Physiol. Rev..

[6] Peers C., Bauer C.C., Boyle J.P., Scragg J.L., Dallas M.L. (2011). Modulation of ion channels by hydrogen sulfide. Antioxid. Redox Signal..

[7] Wu L., Ishigaki Y., Hu Y., Sugimoto K., Zeng W., Harimoto T., Sun Y., He J., Suzuki T., Jiang X. (2020). H_2_S-activatable near-infrared afterglow luminescent probes for sensitive molecular imaging in vivo. Nat. Commun..

[8] Jain S.K., Bull R., Rains J.L., Bass P.F., Levine S.N., Reddy S., McVie R., Bocchini J.A. (2010). Low levels of hydrogen sulfide in the blood of diabetes patients and streptozotocin-treated rats causes vascular inflammation. Antioxid. Redox Signal..

[9] Kamoun P., Belardinelli M.C., Chabli A., Lallouchi K., Chadefaux-Vekemans B. (2003). Endogenous hydrogen sulfide overproduction in Down syndrome. Am. J. Med. Gen..

[10] Li B., Li L., Wang K., Wang C., Zhang L., Liu K. (2017). Ultrasensitive and facile electrochemical detection of hydrogen sulfide in rat brain microdialysate based on competitive binding reaction. Anal. Bioanal. Chem..

[11] Zeng J., Li M., Liu A., Feng F., Zeng T., Duan W., Li M., Gong M., Wen C.Y., Yin Y. (2018). Au/AgI dimeric nanoparticles for highly selective and sensitive colorimetric detection of hydrogen sulfide. Adv. Funct. Mater..

[12] Montoya L.A., Shen X., McDermott J.J., Kevil G.G., Pluth M.D. (2015). Mechanistic investigations reveal that dibromobimane extrudes sulfur from biological sulfhydryl sources other than hydrogen sulfide. Chem. Sci..

[13] Niu L.Y., Chen Y.Z., Zheng H.R., Wu L.Z., Tung C.H., Yang Q.Z. (2015). Design strategies of fluorescent probes for selective detection among biothiols. Chem. Soc. Rev..

[14] Yu F., Han X., Chen L. (2014). Fluorescent probes for hydrogen sulfide detection and bioimaging. Chem. Commun..

[15] Kaur P., Singh K. (2019). Recent advances in the application of BODIPY in bioimaging and chemosensing. J. Mater. Chem. C.

[16] Kowasa T., Mawda H., Kikuchi K. (2015). BODIPY-based probes for the fluorescence imaging of biomolecules in living cells. Chem. Soc. Rev..

[17] Zhang J., Wang N.N., Ji X., Tao Y.F., Wang J.M., Zhao W.L. (2020). BODIPY-based fluorescent probes for biothiols. Chem. Eur. J..

[18] Zhu X.-Y., Wu H., Guo X.-F., Wang H. (2019). Novel BODIPY-based fluorescent probes with large Stokes shift for imaging hydrogen sulfide. Dyes Pigments.

[19] Li Q., Wang Z., Zhao M., Hong Y., Jin Q., Yao S., Zheng C., Quan Y.-Y., Ye X., Huang Z.-S. (2019). A NIR fluorescent probe for the detection and visualization of hydrogen sulfide in colorectal cancer cell. Sens. Actuator B Chem..

[20] Yue J., Tao Y., Zhang J., Wang H., Wang N., Zhao W. (2021). BODIPY-based Fluorescent Probe for Fast Detection of Hydrogen Sulfide and Lysosome-targeting Applications in Living Cells. Chem. Asian J..

[21] Wang R., Gao W., Gao J., Xu G., Zhu T., Gu X., Zhao C. (2019). A forster resonance energy transfer switchable fluorescent probe with H_2_S-activated second near-infrared emission for bioimaging. Front. Chem..

[22] Qian J., Gong D., Teng Z., Wang J., Cao T., Iqbal K., Liu W., Iqbal A., Qin W., Guo H. (2019). 2-Vinylfuran substituted BODIPY H_2_S fluorescent turn on probe based on hydrolysis of furfural and nucleophilic addition of double bond. Sens. Actuator B Chem..

[23] Zhao Q., Yin C.X., Kang J., Wen Y., Huo F.J. (2018). A viscosity sensitive azide-pyridine BODIPY-based fluorescent dye for imaging of hydrogen sulfide in living cells. Dyes Pigments.

[24] Jia Y., Xia L.-J., Chen L., Guo X.-F., Wang H., Zhang H.-J. (2018). A novel BODIPY-based fluorescent probe for selective detection of hydrogen sulfide in living cells and tissues. Talanta.

[25] Zhang J., Ji X., Zhou J., Dong X., Chen Z., Zhao W. (2018). Pyridinium substituted BODIPY as NIR fluorescent probe for simultaneous sensing of hydrogen sulfide/glutathione and cysteine/homocysteine. Sens. Actuator B Chem..

[26] Quan Y.Y., Fan L.N., Shen H.Y., Wu B.N., Kong S.N., Luo Y.S., Huang Z.S., Ye X.X. (2022). A multifunctional BODIPY based fluorescent probe for hydrogen sulfide detection and photodynamic anticancer therapy in HCT116 colon cancer cell. Dyes Pigments.

[27] Wang J., Yu H., Li Q., Shao S. (2015). A BODIPY-based turn-on fluorescent probe for the selective detection of hydrogen sulfide in solution and in cells. Talanta.

[28] Fang T., Jiang X.-D., Sun C., Li Q. (2019). BODIPY-based naked-eye fluorescent on-off probe with high selectivity for H_2_S based on thiolysis of dinitrophenyl ether. Sens. Actuator B Chem..

[29] Zhao C., Zhang X., Li K., Zhu S., Guo Z., Zhang L., Wang F., Fei Q., Luo S., Shi P. (2015). Förster resonance energy transfer switchable self-assembled micellar nanoprobe: Ratiometric fluorescent trapping of endogenous H_2_S generation via fluvastatin-stimulated upregulation. J. Am. Chem. Soc..

[30] Gao M., Wang R., Yu F.B., You J., Chen L.X. (2015). A near-infrared fluorescent probe for the detection of hydrogen polysulfides biosynthetic pathways in living cells and in vivo. Analyst.

[31] Liu K., Liu C., Shang H., Ren M., Lin W. (2018). A novel red light emissive two-photon fluorescent probe for hydrogen sulfide (H_2_S) in nucleolus region and its application for H_2_S detection in zebrafish and live mice. Sens. Actuators B Chem..

[32] Feng S., Xia Q., Feng G. (2019). Iminocoumarin-based red to near-infrared fluorescent turn-on probe with a large Stokes shift for imaging H_2_S in living cells and animals. Dyes Pigments.

[33] Shu W., Zang S., Wang C., Gao M., Jing J., Zhang X. (2020). An endoplasmic reticulum targeted ratiometric fluorescent probe for sensing of hydrogen sulfide in living cells and zebrafish. Anal. Chem..

[34] Wu Q., Yin C., Wen Y., Zhang Y., Huo F. (2019). An ICT lighten ratiometric and NIR fluorogenic probe to visualize endogenous/exogenous hydrogen sulphide and imaging in mice. Sens. Actuator B Chem..

[35] Song X., Wang Y., Ru J., Yang Y., Feng Y., Cao C., Wang K., Zhang G., Liu W. (2020). A mitochondrial-targeted red fluorescent probe for detecting endogenous H_2_S in cells with high selectivity and development of a visual paper-based sensing platform. Sens. Actuator B Chem..

[36] Xia S., Shen J., Wang J., Wang H., Zhou H., Tanasova M. (2018). Ratiometric fluorescent and colorimetric BODIPY-based sensor for zinc ions in solution and living cells. Sens. Actuator B Chem..

[37] Pang Z., Ye H., Ma D., Tu X., Yi L., Xi Z. (2021). A H_2_S-specific ultrasensitive fluorogenic probe reveals TMV-induced H2S production to limit virus replication. ChemBioChem.

[38] Paul N., Sarkar R., Sarkar R., Barui A., Sarkar S. (2020). Detection of hydrogen sulfide using BODIPY based colorimetric and fluorescent on-off chemosensor. J. Chem. Sci..

[39] Gong D.Y., Zhu X.T., Tian Y.J., Han S.C., Deng M., Iqbal A., Liu W.S., Qin W., Guo H.C. (2017). A Phenylselenium-Substituted BODIPY Fluorescent Turn-off Probe for Fluorescence Imaging of Hydrogen Sulfide in Living Cells. Anal. Chem..

[40] Fei Q., Li M.M., Chen J., Shi B., Xu G., Zhao C.C., Gu X.F. (2018). Design of BODIPY-based near-infrared fluorescent probes for H2S. J. Photochem. Photobiol. A Chem..

