# Supplementary material for: A New Ratiometric Fluorescent Probe Based on BODIPY for Highly Selective Detection of Hydrogen Sulfide
Source: Molecules. 2022 Nov 3;27(21):7499. doi: 10.3390/molecules27217499 (PMC9653583; doi:10.3390/molecules27217499)

Supporting Information

A New Ratiometric Fluorescent Probe Based on BODIPY for Highly Selective Detection of Hydrogen Sulfide

Huan Xiang, Tianqing Ye*, Yanbo Li, Yanfei Lin, Dan Liu, Hongwei Zhou, Jianbo Wang*, Lei Li*

College of Biological, Chemical Sciences and Engineering, Jiaxing University, Jiaxing Key Laboratory of Molecular Recognition and Sensing, Jiaxing 314001, China.

E-mail: yetianqing@163.com; wjb4207@mail.ustc.edu.cn; lei.li@mail.zjxu.edu.cn.

Table of Contents

Table S1. The BODIPY-Based fluorescent probes for detection of H_2S. S3- S5

Figure S1. The absorption ratio at 568 nm and 516 nm of probe **BH** (5 μM) toward different concentration of H_2S in aqueous solution of DMSO/PBS (v/v 1/1, pH 7.4). S6

Figure S2. The fluorescence intensity at 568 nm and 516 nm of probe **BH** (5 μM) toward increasing H_2S concentration (0−500 μM) in aqueous solution of DMSO/PBS (v/v 1/1, pH 7.4). S6

Figure S3. Linear relationship of the fluorescence intensity ratio (I_{594nm}/I_{542nm}) of probe **BH** toward H_2S concentration (0−75 μM). S7

Figure S4. ESI-MS spectrum of reaction product of probe **BH** with H_2S. S8

Figure S5. The fluorescence intensity ratios (I_{594nm}/I_{542nm}) of probe BH (5 μM) in the absence (●)or presence (■) of H_2S (500 μM) at various pH values. S9

Copies of ^{1}H and ^{13}C NMR spectra of new compound and probe S10-S12

Reference S13-S14

Table S1. The BODIPY-Based fluorescent probe for detection of H_2S

Probe structure	Response mode	Response time	Detection limit	Reference
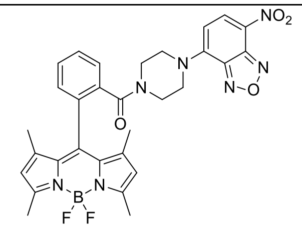	Turn-on<br>520 nm	60 min (100 uM H_2S)	105 nM	37
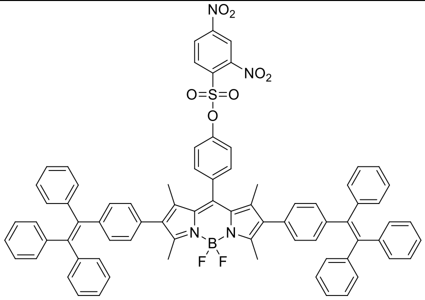	Turn-on<br>520 nm	210 s	6.39 nM	26
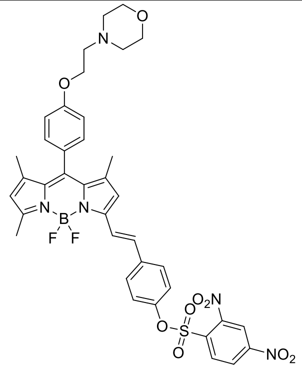	Turn-on<br>587 nm	1 min (50 uM H_2S)	51 μM	20
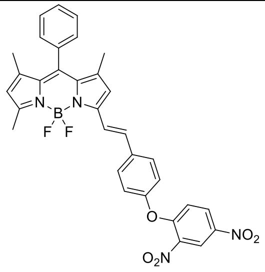	Turn-on<br>592 nm	20 min (100 uM H_2S)	1.27 μM	24
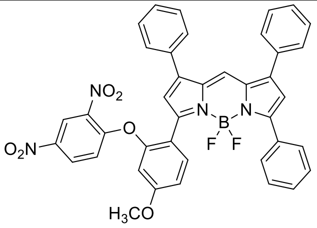	Turn-off<br>615 nm	20 min	0.203μM	28

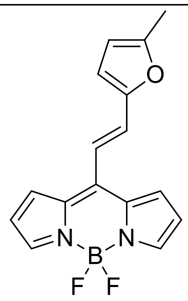	Turn-on<br>496 nm	30 min	99.4 nM	22
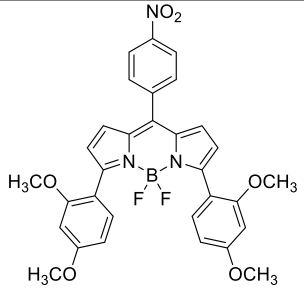	Turn-on<br>627 nm	40 min	1.3 μM	18
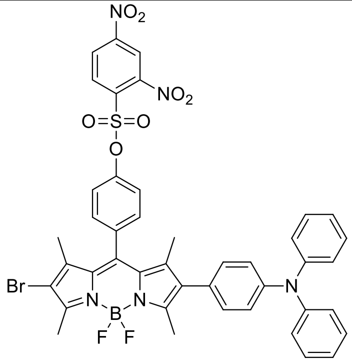	Turn-on<br>716 nm	150 s (150 uM H_2S)	6.74μM	19
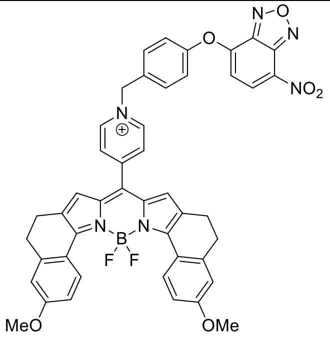	Turn-on<br>685 nm	10 min (100 uM)	1.15 μM	25
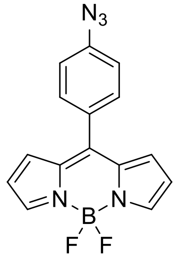	Turn-off<br>516 nm	-	0.17 μM	38

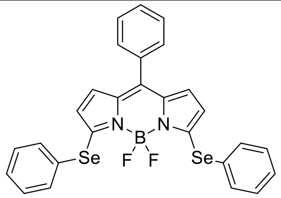	Turn-off<br>610 nm	20 min	2.5 nM	39
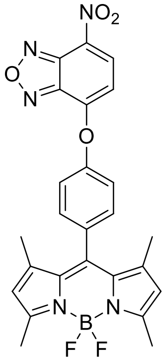	Turn-on<br>510 nm	-	2.6 μM	27
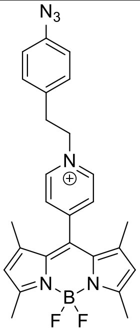	Turn-on<br>515 nm	15 min	2.05 μM	23
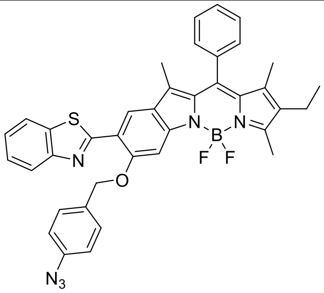	Turn-on<br>700 nm	90 min	46 nM	40
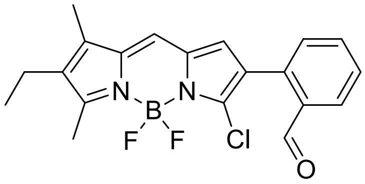	Raticmaric fluorescnece From 542 nm to 594 nm	60 min	3.5 μM	This Work

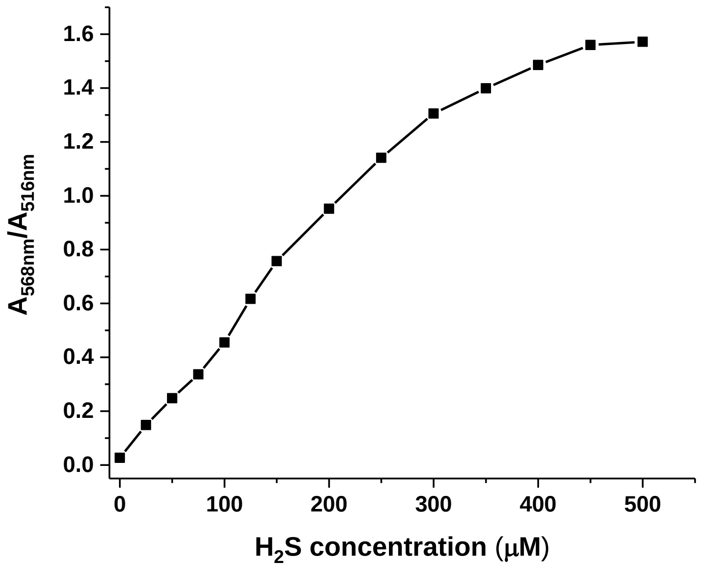

Figure S1. The absorption ratio at 568 nm and 516 nm of probe **BH** (5 μM) toward different concentration of H_2S in aqueous solution of DMSO/PBS (v/v 1/1, pH 7.4), recorded after 60 min.

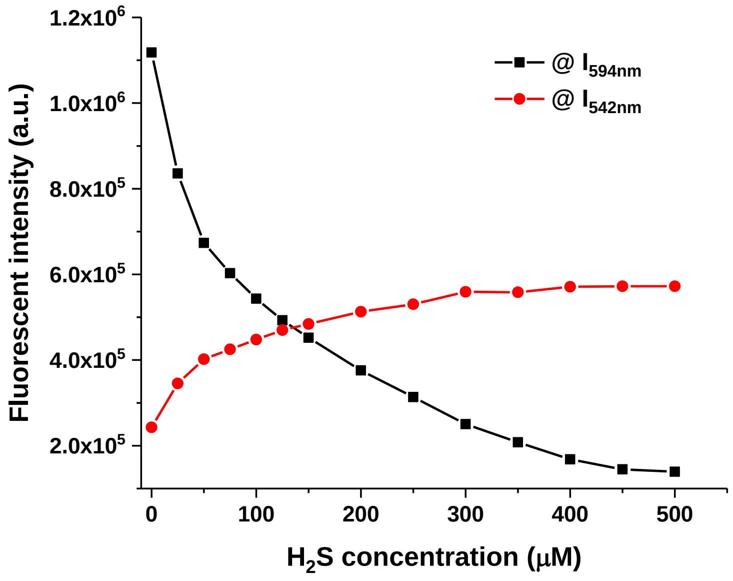

Figure S2. The fluorescence intensity at 568 nm and 516 nm of probe **BH** (5 μM) toward increasing H_2S concentration (0−500 μM) in aqueous solution of DMSO/PBS (v/v 1/1, pH 7.4), recorded after 60 min.

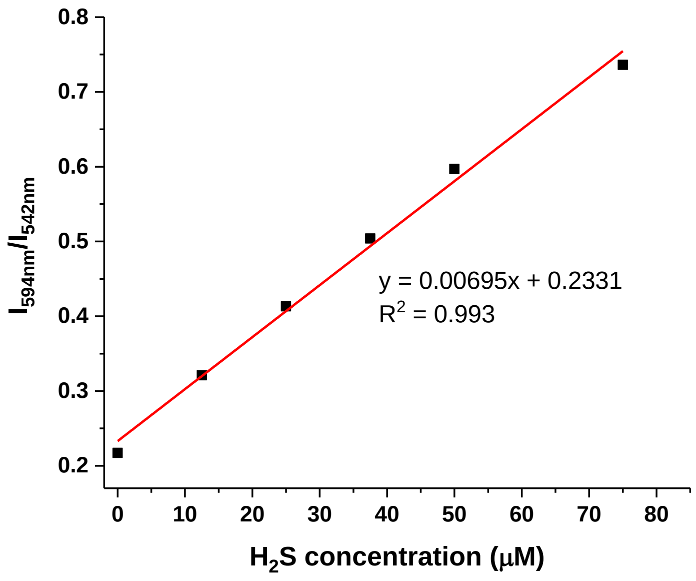

Figure S3. Linear relationship of the fluorescence intensity ratio (I_{594nm}/I_{542nm}) of probe **BH** toward H_2S concentration (0−75 μM), recorded after 60 min.

Display Report

Analysis Info

Analysis Name	D:\Data\2020MS\ZF\0114\bs_GD3_01_691.d	Acquisition Date	1/13/2020 12:03:48 PM
Method	tune_low_200-800_neg-6min.m	Operator	gftang
Sample Name	bs	Instrument / Ser#	micrOTOF 10257
Comment			

Acquisition Parameter

Source Type	ESI	Ion Polarity	Negative	Set Nebulizer	0.4 Bar
Focus	Not active			Set Dry Heater	200 ℃
Scan Begin	100 m/z	Set Capillary	4000 V	Set Dry Gas	4.0 l/min
Scan End	1000 m/z	Set End Plate Offset	-500 V	Set Divert Valve	Waste

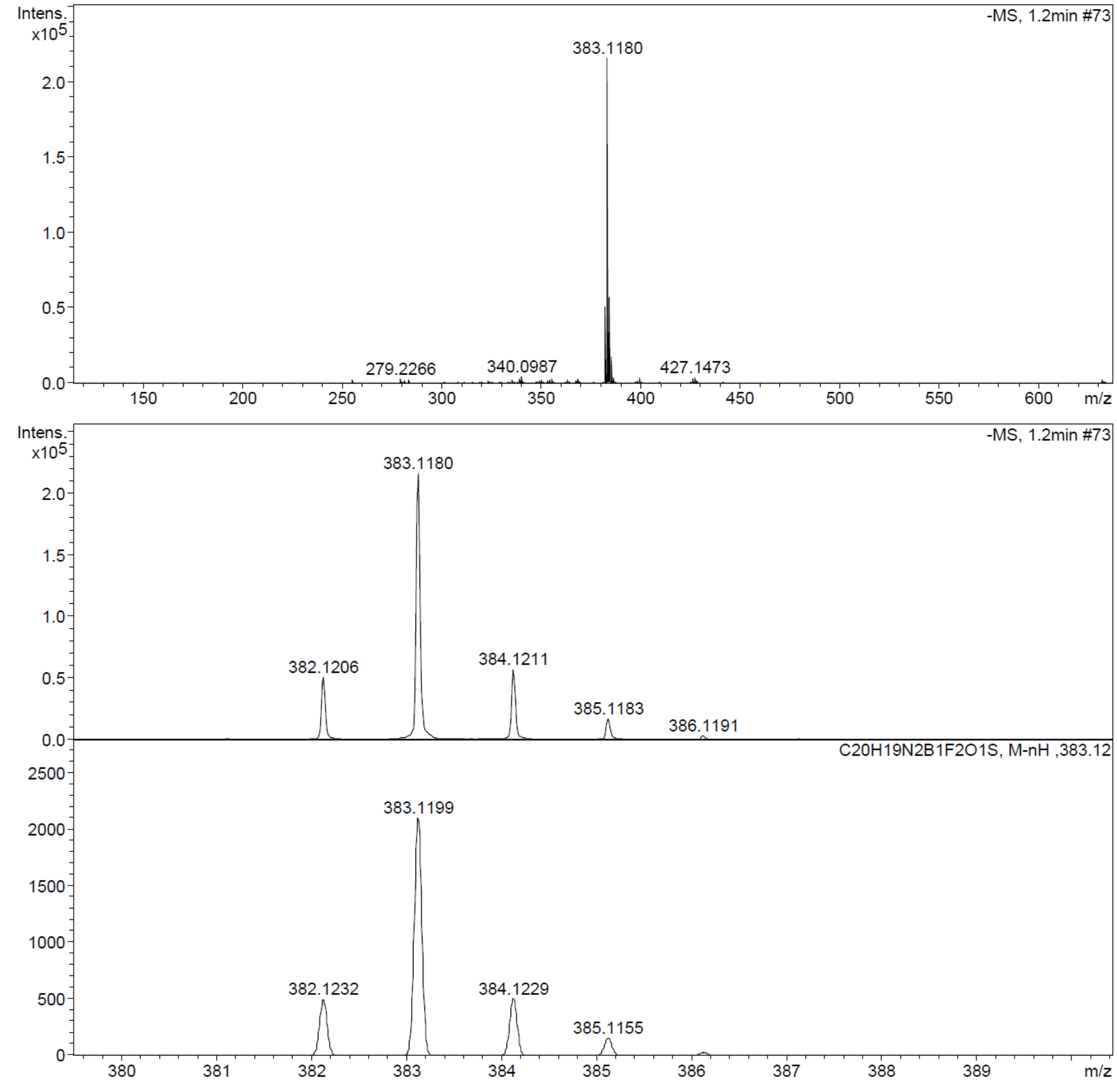

Bruker Compass DataAnalysis 4.0 printed: 1/13/2020 12:11:10 PM Page 1 of 1

Figure S4. ESI-MS spectrum of reaction product of probe **BH** with H_2S.

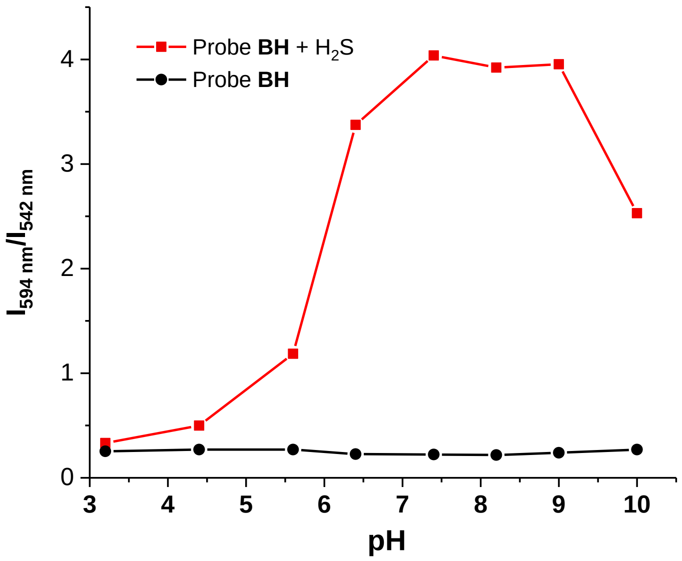

Figure S5. The fluorescence intensity ratios (I_{594nm}/I_{542nm}) of probe **BH** (5 μM) in the absence (●)or presence (■) of H_2S (500 μM) at various pH values, recorded after 60 min.

Copies of ^{1}H and ^{13}C NMR spectra of new compounds

^{1}H NMR spectra (400 MHz) of compound **2** in $CDCl_3$ solution

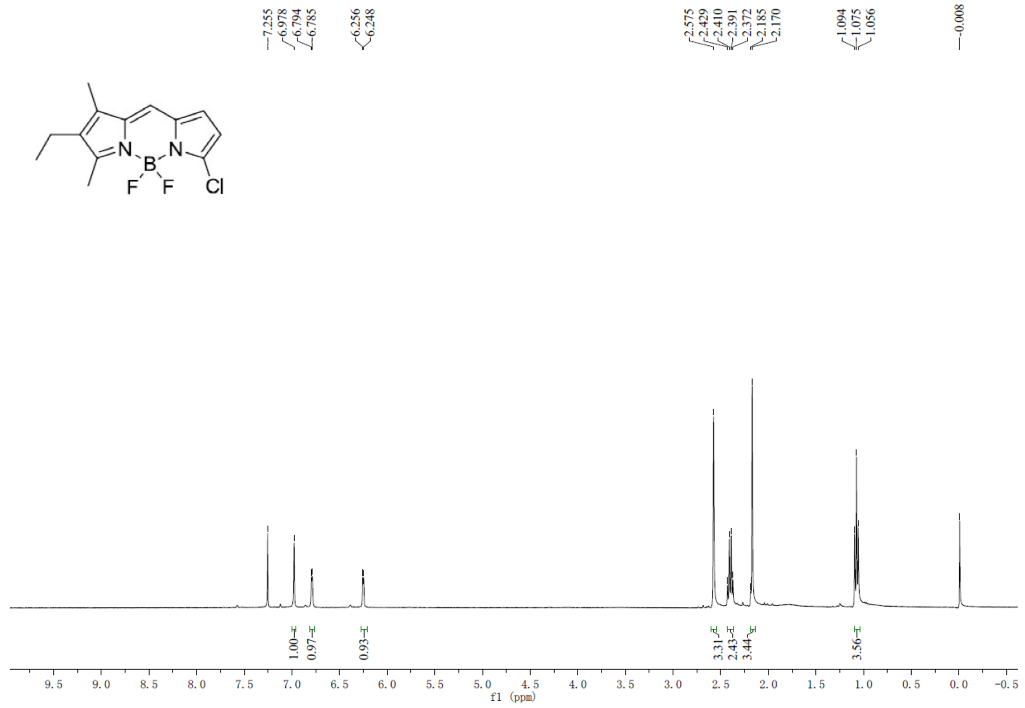

^{13}C NMR spectra (100 MHz) of compound **2** in $CDCl_3$ solution

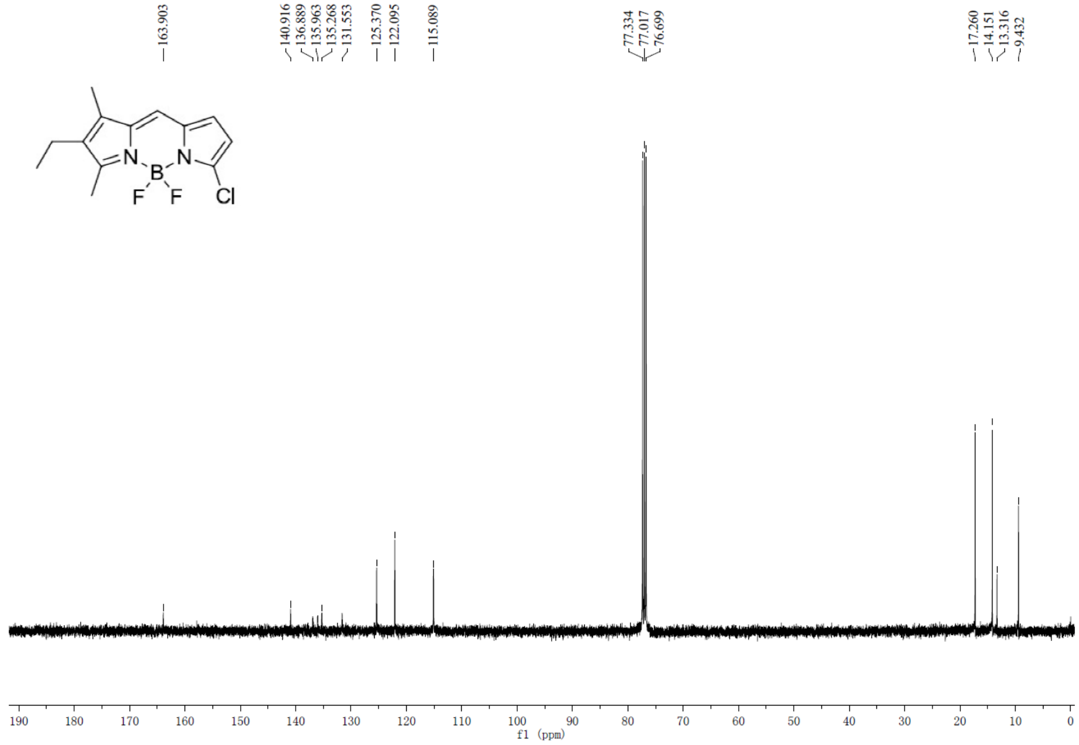

^{1}H NMR spectra (400 MHz) of compound **3** in $CDCl_3$ solution

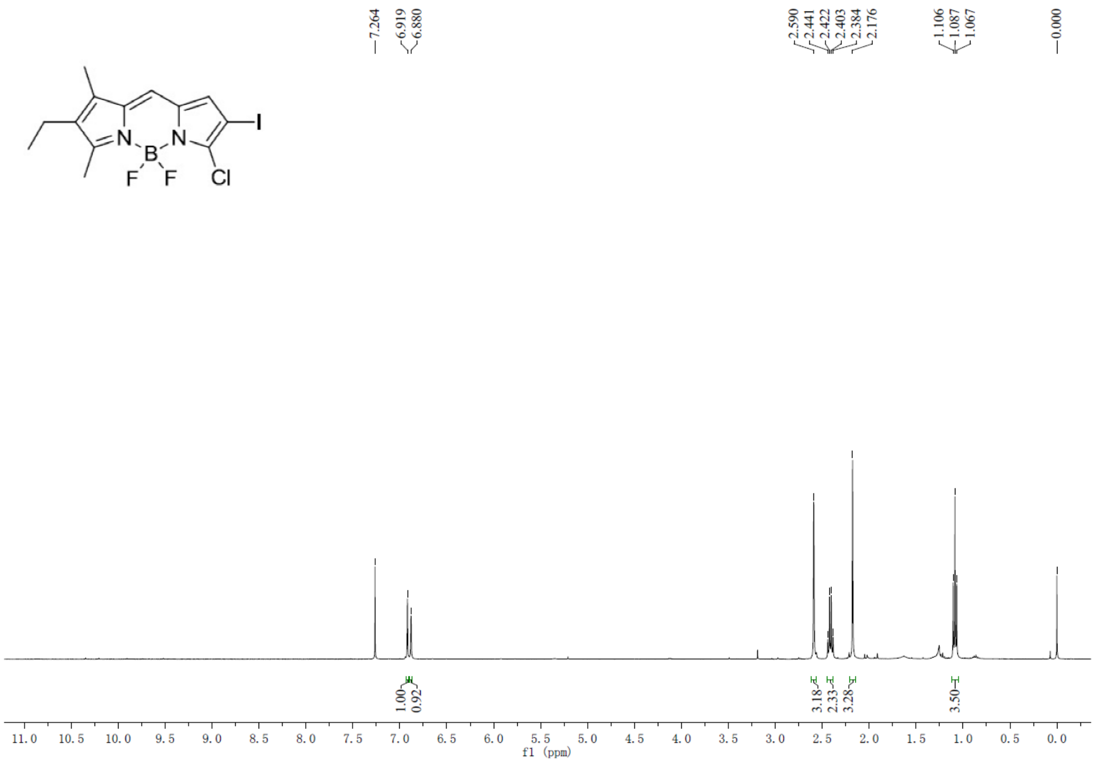

^{13}C NMR spectra (100 MHz) of compound **3** in $CDCl_3$ solution

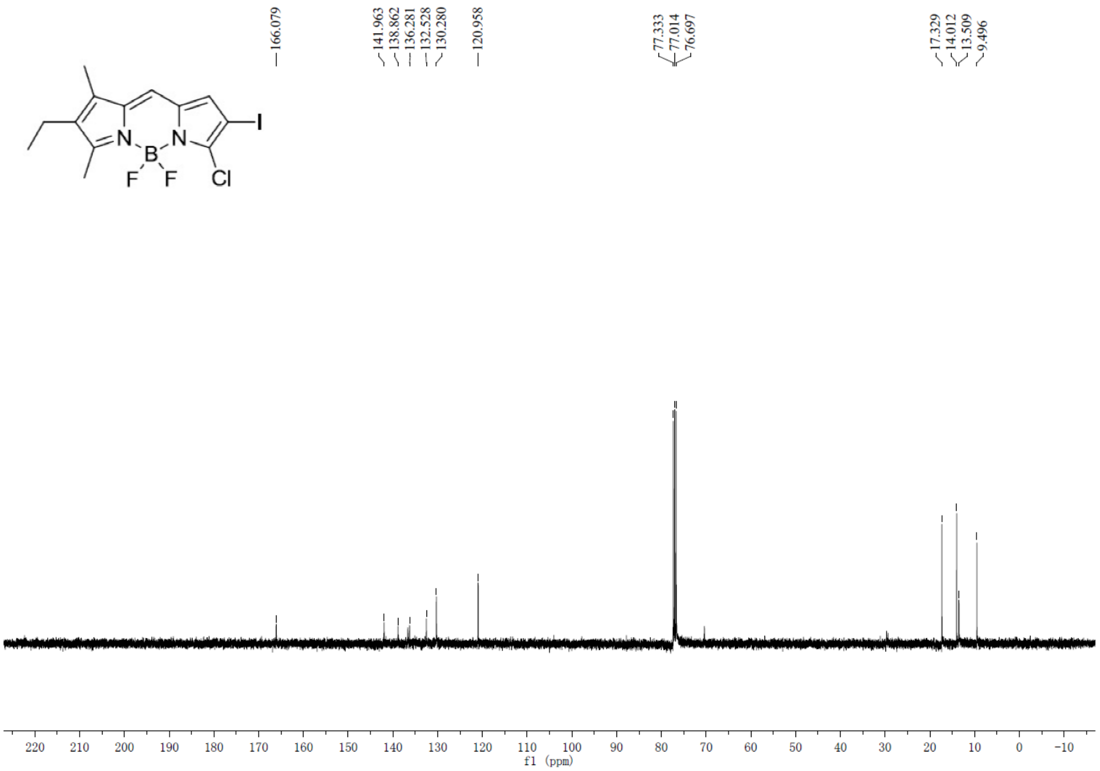

^1H NMR spectra (400 MHz) of probe **HS** in $CDCl_3$ solution

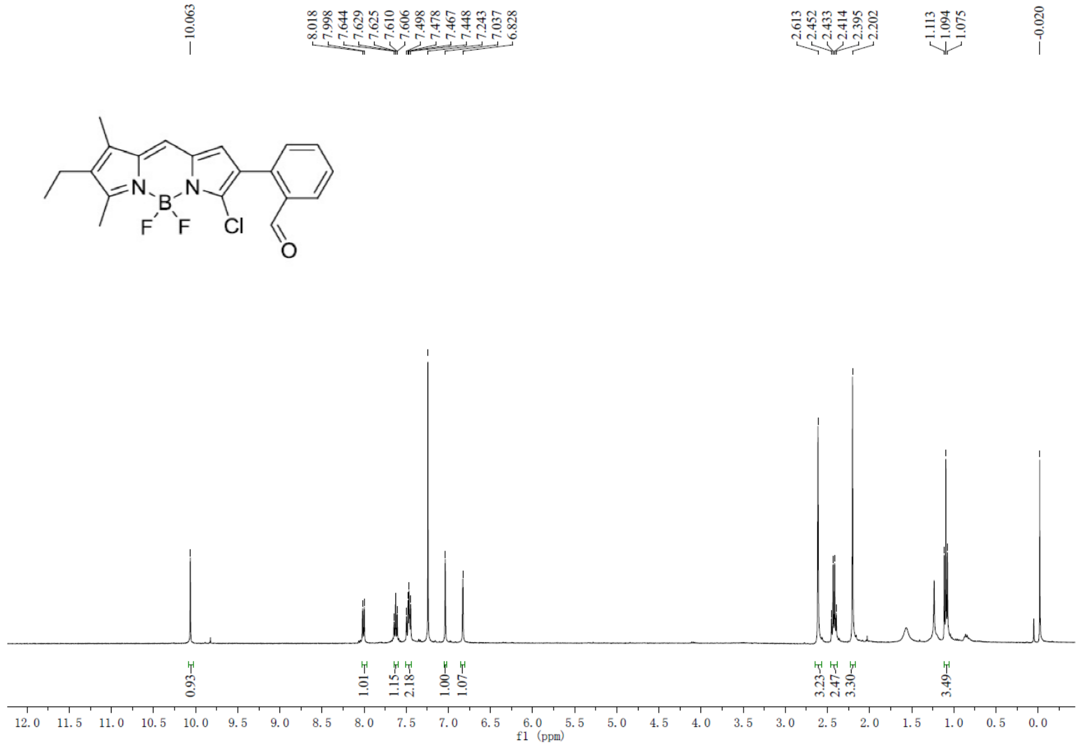

^{13}C NMR spectra (100 MHz) of probe **HS** in $CDCl_3$ solution

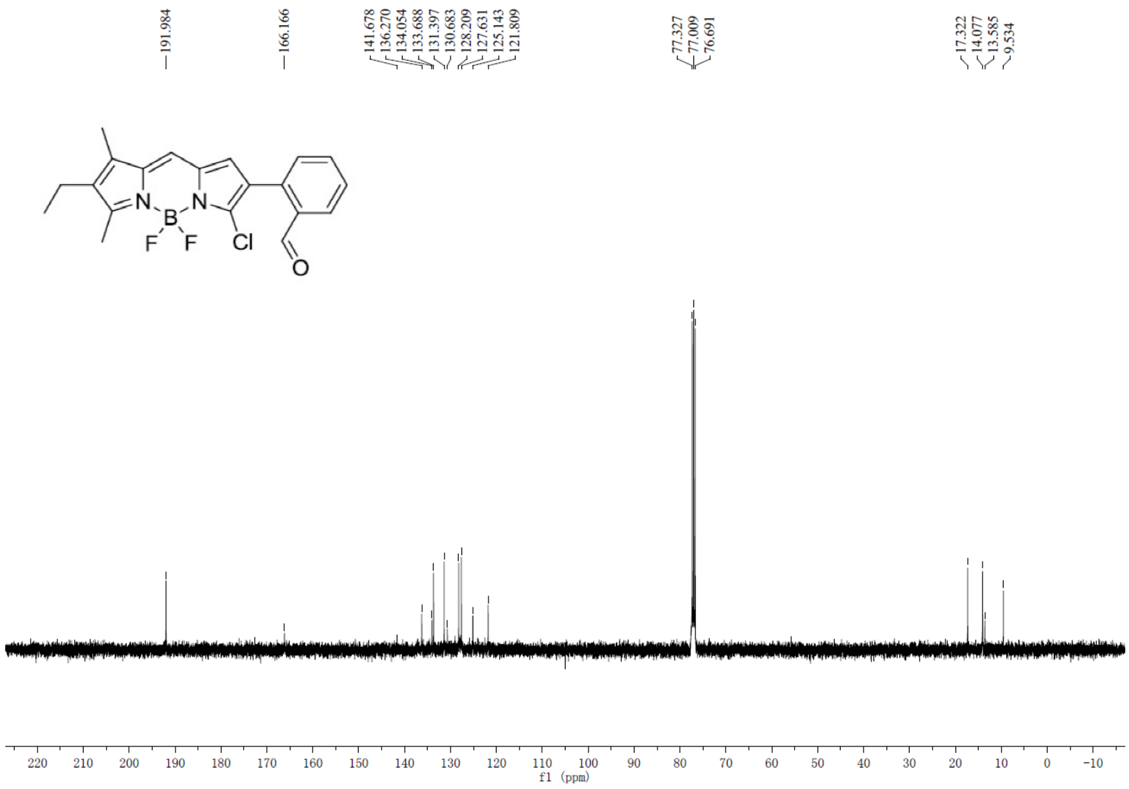

Supplement: Supplementary file 1 [file molecules-27-07499-s001.zip › molecules-1948855-supplementary.pdf]
